# Supplementary material for: Association of early discharge and clinical outcomes following proctectomy for patients with rectal cancer: A NRD analysis
Source: PLoS One. 2026 May 4;21(5):e0348125. doi: 10.1371/journal.pone.0348125 (PMC13138680; doi:10.1371/journal.pone.0348125)
Supplement: Attachment — Submitted filename: Response To Reviewers.docx [file pone.0348125.s002.docx]

**November 20, 2025**

**Revision note for manuscript entitled “Association of early discharge and clinical outcomes following proctectomy for patients with rectal cancer: a NRD analysis”**

**When submitting your revision, we need you to address these additional requirements.**

**1. Please ensure that your manuscript meets PLOS ONE's style requirements, including those for file naming. The PLOS ONE style templates can be found at https://journals.plos.org/plosone/s/file?id=wjVg/PLOSOne_formatting_sample_main_body.pdf and https://journals.plos.org/plosone/s/file?id=ba62/PLOSOne_formatting_sample_title_authors_affiliations.pdf**

 This has been addressed.

**2. We noticed you have some minor occurrence of overlapping text with the following previous publication(s), which needs to be addressed:**

**https://pubmed.ncbi.nlm.nih.gov/38938260/**

**https://www.clinicalkey.com/#!/content/playContent/1-s2.0-S0003497521007013?returnurl=https:%2F%2Flinkinghub.elsevier.com%2Fretrieve%2Fpii%2FS0003497521007013%3Fshowall%3Dtrue&referrer=https:%2F%2Fapi.ithenticate.com%2F**

**In your revision ensure you cite all your sources (including your own works), and quote or rephrase any duplicated text outside the methods section. Further consideration is dependent on these concerns being addressed**

 This has been addressed.

**3. We note that you have indicated that there are restrictions to data sharing for this study. PLOS only allows data to be available upon request if there are legal or ethical restrictions on sharing data publicly. For more information on unacceptable data access restrictions, please see http://journals.plos.org/plosone/s/data-availability#loc-unacceptable-data-access-restrictions.

Before we proceed with your manuscript, please address the following prompts:

a) If there are ethical or legal restrictions on sharing a de-identified data set, please explain them in detail (e.g., data contain potentially identifying or sensitive patient information, data are owned by a third-party organization, etc.) and who has imposed them (e.g., a Research Ethics Committee or Institutional Review Board, etc.). Please also provide contact information for a data access committee, ethics committee, or other institutional body to which data requests may be sent.**

The data utilized in this study is owned by a third-party organization and were derived from the Nationwide Readmissions Database (NRD), which is publicly available upon purchase through the Healthcare Cost and Utilization Project (HCUP). Due to the deidentified nature of the NRD, individual patient data cannot be shared directly by the authors. Access to the NRD is available to eligible researchers through HCUP following data use agreement protocols. HIPAA-compliant, deidentified data can be requested by any researcher at: <https://hcup-us.ahrq.gov/tech_assist/centdist.jsp>

**Please can you confirm whether the authors of your manuscript had to purchase access to the data provided by the HCUP as well, as we require that other researchers would be able to access the data set in the same manner as the authors.**

We confirm that our research lab and its affiliated authors purchased access to the data provided by the HCUP as well.

**b) If there are no restrictions, please upload the minimal anonymized data set necessary to replicate your study findings to a stable, public repository and provide us with the relevant URLs, DOIs, or accession numbers. For a list of recommended repositories, please see
https://journals.plos.org/plosone/s/recommended-repositories. You also have the option of uploading the data as Supporting Information files, but we would recommend depositing data directly to a data repository if possible.**

N/A

**We will update your Data Availability statement on your behalf to reflect the information you provide.**

**4. Please ensure that you refer to Figure 4 in your text as, if accepted, production will need this reference to link the reader to the figure.**

 This has been addressed

**5.** [**Please remove all personal information, ensure that the data shared are in accordance with participant consent, and re-upload a fully anonymized data set.

Note: spreadsheet columns with personal information must be removed and not hidden as all hidden columns will appear in the published file.

Additional guidance on preparing raw data for publication can be found in our Data Policy (https://journals.plos.org/plosone/s/data-availability#loc-human-research-participant-data-and-other-sensitive-data) and in the following article: http://www.bmj.com/content/340/bmj.c181.long.**](http://www.bmj.com/content/340/bmj.c181.long.)

**Reviewer #1: 

Major:
1a. Most critically, it’s not surprising that healthier patients with less extensive disease and comorbidities would be better candidates for early discharge. If the cohorts were truly balanced (comparing similar patient populations), one could draw stronger conclusions from this paper. As currently presented, being discharged early seems to be driven more by an inherently healthier patient than by physician choice to “push the envelope” and expedite a patient’s discharge. The paper would be more useful if framed from the latter. If the patient cohorts were better matched, I would expect that readmissions and complication rates to be similar, not improved.**

**1b. Given the large proportion of laparoscopic proctectomy in the Early group, this would suggest there was clear selection bias that the study may not have granularity to ascertain. I would suggest that the authors perform a secondary/sensitivity analysis either using a propensity-matched analysis (such as inverse probability of treatment weighing or entropy balancing) and/or perform a separate analysis evaluating only patients who underwent laparoscopic surgery**

**1c. Presence of metastatic disease, which was higher in the Routine group, would also suggest that the groups were not balanced and Routine patients had more complex burden of disease, including radiation**

We thank the reviewer for these important comments. We fully agree that the baseline imbalance and selection bias are inherent limitations in retrospective database studies of discharge timing. We have addressed your suggestions and performed entropy balancing to construct a weighted comparison group with closely matched covariate distributions across patient and hospital characteristics. This method has been shown to outperform traditional propensity score matching by improving covariate balance without reducing sample size. After entropy balancing, all standardized mean differences were <0.1 (Fig 3), indicating excellent balance of surgical approach, comorbidity burden, and metastatic disease. Importantly, the associations between early discharge and decreased readmissions, LOS, and hospitalization costs remained statistically significant after weighting, suggesting that findings were not solely driven by healthier baseline profiles.  **2. Given the inability to know the timing of diagnoses, how are the authors able to assess whether a complication prevented a patient from being discharged Early? It looks like the authors looked at complications at the readmission, but didn’t make any comparisons during the index hospitalization. This would obviously skew who would be Early**

We thank the reviewer for highlighting this concern. The NRD does not provide time-stamped intra-hospital complication data (i.e whether complications occurred before or after operation). For this reason, we could not accurately use complications during index admission to determine impact on discharge after proctectomy.

**3a.** **How did the authors define a “major complication?” As above, depending on what it is, it may delay a discharge and cause selection bias. The authors should consider performing separate analysis removing patients who underwent major complications during the index hospitalization

3b. It is also possible that a complication could cause a significant increase in the LOS. I suggest that the authors omit patients who stayed significantly longer than expected. This should improve the homogeneity of their cohort**

We thank the reviewer for this suggestion. In the NRD, complications are captured using ICD-10 codes and grouped into validated categories (cardiac, respiratory, infectious, renal, thromboembolic, and transfusion-related). In our study, major complications included: cardiac, respiratory, infectious, renal, thromboembolic, transfusion-related, and strokes. However, the NRD does not provide timestamped information for complications occurring during index hospitalization which prevents us from reliably excluding patients whose early postoperative complication may have directly impacted their discharge timing. But because severe postoperative complications often do result in prolonged and clinically atypical lengths of stay, we excluded patients with an index LOS > 9 days which corresponded to the 90^th^ percentile to decrease the heterogeneity of the cohort.

“Finally, patients with a length of stay greater than 9 days (90^th^ percentile for length of stay) were excluded to decrease the heterogeneity of the cohort.”

**4. What were the reasons for readmissions for those requiring readmission? The non-elective readmission rate for both groups is relatively high. I’m not sure about the clinical significance of reporting mortality on the readmission given how low this value is. Discussing needing blood products upon readmission may not be a clinically helpful metric either. Critically, did any of them develop leaks and how did this compare between groups? This would be a more clinically-relevant reason for readmission to report and would be more important for the readership**

We thank the reviewer for this valuable suggestion. Unfortunately, the NRD does not provide granular clinical details regarding the specific cause of readmission (e.g anastomatic leak, abscess, etc.) which limits our ability to directly compare clinically meaningful etiologies such as leaks across early and routine discharge groups.

Regarding the readmission outcomes we reported, the non-elective readmission rate is indeed relatively high for both cohorts, which we believe reflects the overall complexity of proctectomy patients included in the NRD sample. Although readmission mortality was low, we reported it for completeness and transparency.

Similarly, the need for blood transfusion during readmission is available within the NRD as a coded variable but does not reliably distinguish between clinically meaningful etiologies and was included for completeness.

**5. Are the authors able to specify if a patient received a LAR or APR? What about how many ended up getting ileostomies? Obviously extent of operation would determine who could be a candidate for early discharge**

We appreciate this question. The NRD does not contain operative detail separating LAR from APR. However, ileostomy creation is captured and was incorporated into both the unadjusted and adjusted models. Ileostomy creation did not alter the likelihood of early discharge (AOR 1.03; 95% CI 0.95 , 1.12; Table 2). We have clarified this in Results.

**6. The authors should mention more clearly in their limitations that patient selection criteria is unable to be accounted for. This is the largest limitation of the study and should be mentioned explicitly**

We agree and have strengthened this statement in the limitations.

“Specifically, patient selection criteria for early discharge such as surgeon preference, patient functional status are not captured in the NRD and could not be accounted for. These unmeasured factors represent an important source of potential selection bias.”

**Minor:
- What proportion of patients were robotic-assisted?**

We thank the reviewer for this question. Robotic approach identifiers were not reliably available in the NRD because ICD-10-PCS differentiates between laparoscopic and open approaches but does not consistently capture robotic assistance.

**- Are the authors able to specify where the rectal cancer metastasis was located? Were there patients that may have had concomitant liver resection?**

We appreciate this point. The NRD does not specify the anatomic site of metastatic disease. Additionally, only 3 patients in the dataset had liver resections during index hospitalization.

**- How many patients had a history of radiation?**

About 34.6% of our cohort had prior radiation based on the NRD ICD-10 codes. However, these are rarely used and unreliable as it does not indicate location and further granular details regarding the radiation are not included.

**- How did the authors calculate cumulative LOS in the results section? It would be obvious that the LOS in Early would be shorter than that of Routine in the index hospitalization. This should be better defined in the methods section**

Thank you for the opportunity to clarify how 30-day cumulative LOS was calculated. Because the NRD captures all readmissions to participating hospitals within the same calendar year, we constructed a cumulative LOS variable by summing the index hospitalization LOS with any subsequent readmission occurring within 30 days of discharge. For patient with multiple readmissions, we summed the LOS of the index admission, the first readmission, and the LOS of a second readmission only when the second readmission also fell within the 30-day window.

We have clarified this in the methods.

“Cumulative length of stay (LOS) variable was constructed by summing the index hospitalization LOS with any subsequent readmission occurring within 30 days of discharge. For patient with multiple readmissions, we summed the LOS of the index admission, the first readmission, and the LOS of a second readmission only when the second readmission also fell within the 30 day window.”

**- Why do the authors think that early patients had more cardiac complications? This is mentioned in the results and not addressed afterwards**

We thank the review for this question. We believe the difference was most likely an artifact driven by very low event count. However, we found cardiac complications to no longer be significant after entropy balancing.

**- The authors should further discuss the discrepancy of Turrentine study vs their own as opposed to just acknowledging this paper as contrary to their findings**

Thank you for this comment. We appreciate the opportunity to clarify our comparison with the Turrentine study. We included it as an example of a study that corroborated our findings that early discharge was associated with lower rates of unplanned readmissions. Thus, there were no meaningful discrepancy between the two studies.

**- The authors selected only a few co-morbidities in Table 1. I would encourage the authors to include and adjust for additional co-morbidities such as diabetes, hypertension, chronic lung disease, chronic kidney disease. It’s not clear why the authors chose to report neurologic disorders which should not be clinically-relevant in this patient population**

We thank the reviewer and have updated Table 1 to include diabetes, hypertension, chronic lung disease, and CKD.

Neurological disorders were included in our comorbidity adjustment because, although they may not appear directly related to proctectomy, they meaningfully affect perioperative risk, functional status, and discharge disposition. Patients with underlying neurological conditions such as prior stroke, neuromuscular disease, or cognitive impairment may influence length of stay, timing of discharge, and likelihood of readmission, which are central outcomes in our study.

**- In Table 1, the authors should avoid casual language such as “lap” or “comp”**

All casual abbreviations have been revised to their full terminology throughout the manuscript.

**- Although mentioned in the figure, the text should also clearly state that patients with IBD were omitted**

We agree and added explicit text in the methods.

“Similarly, patients with a diagnosis of Ulcerative Colitis or Crohn’s disease were not included for analysis.”

**- Typo on figure 2 should read “laparoscopic”**

Thank you for this remark. The error has been corrected.

**Reviewer #2: 


Introduction
1a. The introduction is solid and highlights the gaps in existing literature. Although authors mention 3 days as the cutoff to define early discharge, most of the quoted literature pertain to colonic procedures or do not distinguish between colonic and rectal procedures. Given the more complex nature of proctectomies, the cutoff for early discharge after proctectomy is debated in the literature. The introduction could be further strengthened by explaining the rationale for choosing 3 days as cutoff to define early discharge specifically after proctectomy, quoting definitions and literature that are specific to proctectomy.**

Thank you for this insightful comment. We agree that proctectomy is generally considered to be more complex than colectomy, and therefore the definition of “early discharge” warrants careful justification. Although much of the existing literature defines early discharge using a 3-day threshold in colorectal surgery broadly, several studies report that median LOS for proctectomy typically ranges from 5-6 days, which is comparable to the 4-7 days median LOS commonly reported for colectomy and supports the usage of a similar 3-day cutoff. We have clarified this rationale in the introduction and strengthened the justification with literature specific to proctectomy.

“Although proctectomy is generally considered to be more complex than colectomy, reported median lengths of stay for proctectomy (5-6 days) overlap with the 4-7 days commonly reported for colectomy, supporting the use of a similar 3-day threshold to identify patients discharged substantially earlier than contemporary practice patterns[8].”

**Methods
-There are several components of the STROBE checklist that are missing, including sample size calculation, plans to address potential confounding, how missing data was handled, etc.**

We appreciate this comment from the reviewer. A formal sample size calculation was not performed because our study utilized the NRD, a large administrative dataset with a fixed annual sample determined by the database design. The analytic cohort size reflects all eligible patients meeting inclusion criteria.

For our approach to confounding variables, we adjusted for a comprehensive set of demographic, comorbidity, procedural, and hospital-level variables. In addition, we used survey-weighted analyses to ensure national representativeness and correct variance estimation. Upon revision request, we also applied entropy balancing to further improve covariate balance between our cohorts.

For missing data, we clarified in the methods section that “missing key variables such as age, sex, and mortality were excluded.”

**-Were diagnosis codes for rectal cancer used in defining the study cohort? If so, what diagnosis codes were used? They should be listed, similar to how proctectomy codes were listed. From the flow diagram, it seems like UC or Crohn’s diagnosis was an exclusion factor. Did you also exclude patients who underwent proctectomy for other diagnoses other than cancer like rectourethral/vaginal fistula, proctitis, rectal trauma, rectal prolapse, FAP etc.? And did you exclude patients who underwent concurrent colectomy (i.e. proctocolectomy for IBD, FAP). More detail re: how the study cohort was defined is needed.**

We thank the reviewer for this important clarification request. We now explicitly list rectal cancer ICD-10 codes C20 which was used to identify patients with rectal cancer. Only patients with a primary diagnosis of rectal cancer who underwent proctectomy were included in the study cohort.

With respect to other indications, we did not specifically exclude patients who had concurrent diagnoses in addition to rectal cancer (i.e proctitis, fistula, rectal trauma, etc…) as long as rectal cancer was present. Our intention was to capture all patients undergoing proctectomy in the setting of rectal cancer, regardless of additional secondary diagnoses. Similarly, we did not exclude patients who underwent concurrent proctocolectomy if they also carried a rectal cancer diagnosis. We have clarified these points in the limitations.

“Although we restricted our cohort to patients with a diagnosis of rectal cancer, we did not distinguish cases performed for concurrent benign indications or concurrent proctocolectomy, which may introduce additional heterogeneity in surgical complexity and postoperative recovery.”

**The authors mention that elastic net regularization method to select model covariates. Did the authors also add clinically important covariates that were selected a priori, in addition to statistically selected covariates? Patients who are discharged early likely have several key underlying differences in patient, disease, and treatment-associated variables that need to be considered**

We appreciate this suggestion. Indeed, certain clinically essential variables (age, sex, metastatic disease, surgical approach, hospital volume, insurance type, comorbidities) were included in our multivariable models regardless of elastic net selection.  **Results
-The flow diagram needs more detail on how patients were included and excluded according to diagnosis codes, as outlined above**

Thank you for pointing this out. We agree that transparent cohort construction is essential. While we considered adding all diagnosis-based inclusion and exclusion criteria to the flow diagram, doing so resulted in an overly crowded and visually unclear figure. For this reason, we kept the diagram focused on the major steps of cohort refinement. However, in response to the request, we have expanded the methods section to clearly detail diagnoses and respective codes used for inclusion and exclusion.

**-Presence of stoma (whether temporary diverting loop ileostomy or colostomy) is a major factor to consider when thinking about early discharge after proctectomy. Patients with new stoma need additional teaching and closer monitoring prior to discharge – was there any effort made to define which patients had concurrent stoma creation? This is an important variable to capture and report, as well as include in the statistical model**

We agree and have incorporated ileostomy creation into both cohort descriptions and adjusted analyses. As noted earlier, ileostomy creation did not impact the likelihood of early discharge in our cohort: (AOR 1.03; 95% CI 0.95 , 1.12; Table 2).

**-The authors should describe which clinical factors were adjusted for in the final analysis**

We thank the reviewer and have included a comprehensive statement summarizing included adjustment variables:

“Subsequently, multivariable logistic regression models were used to assess factors independently associated with in-hospital mortality and perioperative complications, adjusting for patient and hospital characteristics including: patient demographics, comorbidities, insurance type, hospital volume, and surgical approach.”

**-What about anastomotic leak? Was this captured in the database? There are diagnosis codes for anastomotic leaks that have been used in previous studies using the NRD database – as this is a major complication specific to proctectomy, effort should be made to capture and report this in the most accurate way possible.**

We thank the reviewer for this valuable suggestion. Unfortunately, the NRD does not provide granular clinical details regarding complications such as anastomotic leaks. Any attempts at characterizing it would be unreliable as there are no ICD-10 codes exclusively specific for anastomotic leaks. We do agree that its characterization would have been clinically critical for our study.

**Discussion
-The discussion is well written, with detailed description of strengths and weaknesses of the paper, as well as future directions and relevance of the paper in clinical setting.**

We greatly appreciate the reviewer for the positive feedback.
