# Supplementary material for: Association of early discharge and clinical outcomes following proctectomy for patients with rectal cancer: A NRD analysis
Source: PLoS One. 2026 May 4;21(5):e0348125. doi: 10.1371/journal.pone.0348125 (PMC13138680; doi:10.1371/journal.pone.0348125)
Supplement: Attachment — Submitted filename: Early Discharge Reviewer Specific Comments V3.docx [file pone.0348125.s003.docx]

**Reviewer #1: I thank the authors for addressing some of my concerns. The manuscript is improved but requires additional revisions. I have several questions and/or concerns:

1. While it is true that timing of complication cannot be ascertained in their dataset, patients who had one or multiple complications may have still increased their LOS when they otherwise would not have if it was a routine operation. I strongly suggest that the authors perform a subgroup analysis omitting their complications of interest and seeing if this changes their results**

We thank the reviewer for this insightful suggestion. To address the potential confounding effect of complications on LOS, we performed a subgroup analysis restricted to patients without complications during the index admission. Results are quoted below and reflected in the manuscript’s new version:

“subgroup analysis restricted to patients without complications during the index admission, early discharge remained independently associated with reduced odds of non-elective readmission within 30 days (AOR 0.79, 95% CI 0.70 , 0.88), less frequent infectious (AOR 0.80, 95% CI 0.67 , 0.96) and blood transfusion complications (AOR 0.68, 95% CI 0.47 , 0.99), and reduced cumulative 30-day length of stay (β -2.7, 95% CI -2.8 , -2.6) and hospitalization costs (β -$5,200, 95% CI $-7,800 , -2,500) (Table 5).”

2. **There is a difference between completeness and clinical relevance. This data regarding readmission related death/transfusion should be moved to a supplemental table. If it’s not revisited by the authors in the discussion, it’s not worth including in the main body text**

We appreciate this important distinction. In response, we have removed blood transfusion from the tables and result section as we don’t refer to it in the discussion. However, as the lack of increased mortality associated with early discharge is clinically important to our findings, we have succinctly referenced it in the discussion section as seen below:

“Importantly, we noted that early discharge did not alter in-hospital mortality during readmission, suggesting that expedited discharge does not compromise patient safety.”

3**. I disagree regarding the point of unable to assess location of metastatic disease. Please see:** <https://www.icd10data.com/ICD10CM/Codes/C00-D49/C76-C80/C78-/C78.7>

Thank you for this correction and for highlighting this. We have acknowledged these restrictions of our project in our methods and limitations as follows:

Methods:

“Metastatic disease was similarly identified using ICD-10-CM: C77, C78, C79, C80 and modeled as a composite variable to reflect the overall burden of advanced malignancy.”

Limitations:

“Although ICD-10 coding allows for identification of specific metastatic sites, site-specific stratification was not performed in the present study, as our primary aim was to account for the presence of metastatic disease as a marker of systemic disease severity.”

4. **Similarly, you should be able to differentiate APR vs LAR. Obviously APR involves a perineal resection that a LAR does not. I suggest the authors query there codes for these codes:** [**https://cdn-links.lww.com/permalink/prs/d/prs_144_5_2019_09_26_kung_prs-d-18-02422_sdc1.pdf**](https://cdn-links.lww.com/permalink/prs/d/prs_144_5_2019_09_26_kung_prs-d-18-02422_sdc1.pdf)

We thank the reviewer for this thoughtful suggestion. We carefully evaluated the use of ICD-10-PCS codes to differentiate APR from LAR.

In our study, we identified proctectomy using the ICD-10-PCS codes ODTP0ZZ (open) and ODTP4ZZ (laparoscopic), which have been previously validated and specifically correspond to abdominoperineal resection of the rectum. While additional ICD-10-PCS codes exist that may capture other rectal resections, including those suggested, several are categorized as “other” or represent procedures such as bypass operations which raises the concern for potential misclassification and heterogeneity. Because of this, we elected to use a more specific coding strategy to ensure cohort consistency.

5. **The authors should also be able to evaluate for readmissions related to leaks:** <https://icdcodes.ai/diagnosis/anastomotic-leak/documentation>

We appreciate this important suggestion. We have included leak-related complications during readmission as suggested. Please see cited results below:
 “Additionally, *Early* less frequently developed infectious (21.5 vs 27.5%, p<0.01), or anastomotic leak complications (1.0 vs 4.9%, p<0.001, Table 3) during non-elective readmission.”

6. **The robotic assistance codes are here:** [**https://www.icd10data.com/ICD10PCS/Codes/8/E/0/W**](https://www.icd10data.com/ICD10PCS/Codes/8/E/0/W)

We thank the review for this suggestion. We have now incorporated these codes to identify robot-assisted procedures. See result as cited below:
“Finally, *Early* was more likely to undergo a laparoscopic proctectomy (60.9 vs 39.0%, p<0.001) and an Ileostomy creation (43.7 vs 37.8%, p<0.001), but less likely to undergo robotic assistance (14.2 vs 25.7%, p<0.001) and less likely to have a history of radiation (33.3 vs 39.9%, p<0.001).”

**7. History of radiation should be reported in the tables for completeness**

We agree and have now included history of radiation therapy in the baseline characteristics table for completeness and transparency.

“Finally, *Early* was more likely to undergo a laparoscopic proctectomy (60.9 vs 39.0%, p<0.001) and an Ileostomy creation (43.7 vs 37.8%, p<0.001), but less likely to undergo robotic assistance (14.2 vs 25.7%, p<0.001) and less likely to have a history of radiation (33.3 vs 39.9%, p<0.001).”

**Reviewer #2: Thank you to the authors for incorporating our feedback and addressing concerns.
We appreciate the explanations provided regarding sample size, missing data handling, diagnostic codes**

**Regarding Reviewer 1's request for differentiating LAR from APR - there are NRD codes that can allow for differentiation between the two, using ICD codes.**

**There are also ICD codes available to characterize complications such as leak and abscess. Although as authors mentioned, there are no ICD codes exclusively specific for anastomotic leaks, there are codes available to infer a leak (i.e. Disruption of internal surgical wound, Peritoneal abscess, Fistula of intestine). You could use these in combination of procedure codes (i.e. re-laparotomy, lavage, percutaneous drainage etc.) to increase specificity for detecting the complication. This could be a helpful reference to address above issues (using NRD): https://link.springer.com/article/10.1007/s00464-021-08923-y**

We thank the reviewer for reinforcing these important methodological considerations and have addressed each one of them as seen above under reviewer 1.

We again thank the reviewers for their thoughtful and constructive feedback and are more than happy to revise it further as needed for it to be suitable for publication.

Much appreciated!
